# Haiku: New paradigm for the reverse genetics of emerging RNA viruses

**DOI:** 10.1371/journal.pone.0193069

**Published:** 2018-02-13

**Authors:** Thérèse Atieh, Miriam Diala El Ayoubi, Fabien Aubry, Stéphane Priet, Xavier de Lamballerie, Antoine Nougairède

**Affiliations:** UMR "Émergence des Pathologies Virales" (EPV: Aix-Marseille Univ–IRD 190 –Inserm 1207 –EHESP–IHU Méditerranée Infection), Marseille, France; The Scripps Research Institute, UNITED STATES

## Abstract

Reverse genetics is key technology for producing wild-type and genetically modified viruses. The ISA (Infectious Subgenomic Amplicons) method is a recent versatile and user-friendly reverse genetics method to rescue RNA viruses. The main constraint of its canonic protocol was the requirement to produce (*e*.*g*., by DNA synthesis or fusion PCR) 5' and 3' modified genomic fragments encompassing the human cytomegalovirus promoter (pCMV) and the hepatitis delta virus ribozyme/simian virus 40 polyadenylation signal (HDR/SV40pA), respectively. Here, we propose the ultimately simplified "Haiku" designs in which terminal pCMV and HDR/SV40pA sequences are provided as additional separate DNA amplicons. This improved procedure was successfully applied to the rescue of a wide range of viruses belonging to genera *Flavivirus*, *Alphavirus* and *Enterovirus* in mosquito or mammalian cells using only standard PCR amplification techniques and starting from a variety of original materials including viral RNAs extracted from cell supernatant media or animal samples. We also demonstrate that, in specific experimental conditions, the presence of the HDR/SV40pA is not necessary to rescue the targeted viruses. These ultimately simplified "Haiku" designs provide an even more simple, rapid, versatile and cost-effective tool to rescue RNA viruses since only generation of overlapping amplicons encompassing the entire viral genome is now required to generate infectious virus. This new approach may completely modify our capacity to obtain infectious RNA viruses.

## Introduction

The potential risk to public health posed by emerging viruses have triggered a global effort to study them at the molecular level and develop effective prevention and control strategies [1–5]. Accordingly, significant advances in the development of reverse genetics methods have been proposed in recent years, allowing narrowing the timeframe between the emergence of novel viral pathogens and the availability of reverse genetics systems [6, 7].

In particular, the recent ISA (infectious subgenomic-amplicons) method is a bacterium-free reverse genetics technology, which does not require any cloning step and has been developed to facilitate the study of single-stranded positive-sense RNA viruses [8]. Briefly, the concept of ISA is the production by PCR of overlapping non-infectious subgenomic DNA fragments that encompass the entire viral genome. These overlapping subgenomic amplicons, which can be increased up to ten, are supposed to recombine spontaneously upon transfection into permissive cells [8]. The 5’-extremity of the first fragment and the 3’-extremity of the last fragment are flanked respectively by the human cytomegalovirus promoter (pCMV) and the hepatitis delta virus ribozyme followed by the simian virus 40 polyadenylation signal (HDR/SV40pA). These elements are required to allow the transcription of the viral genomic RNA in susceptible cell, which in turn triggers viral replication. This approach has been applied successfully in mammalian and mosquito cells using a wide range of wild-type and genetically modified single-stranded positive-sense RNA viruses belonging to genera *Flavivirus*, *Enterovirus* and *Alphavirus* [8–11]. An ISA-derived method called ISA-lation has also been proposed to recover infectious viruses directly from nucleic acids-derived clinical/animal samples [12]. In these methods (*i*.*e*. ISA and ISA-lation), the viral genome is amplified in overlapping DNA fragments (usually 3, named pCMV-I, II and III-HDR/SV40pA). However, the most arduous and time-consuming part of these procedures relies in production of the terminal fragments with pCMV and HRR/SV40pA sequences. This part requires therefore either a fusion PCR with pCMV and HDR/SV40pA sequences and/or a *de novo* DNA synthesis. To avoid these pitfalls and to significantly shorten the time required to develop a reverse genetics system for an emerging virus, we investigated here the possibility to provide the pCMV and HDR/SV40pA sequences as separate amplicons and the actual requirement for providing an HDR/SV40pA sequence at the 3’-extremity of the last DNA fragment. We thus propose a reverse genetics method with an ultimately simplified design that we called, the "Haiku" ISA design, with reference to the extreme simplicity but extensive meaning of the Japanese Haiku poems.

## Results and discussion

**Fig 1:**
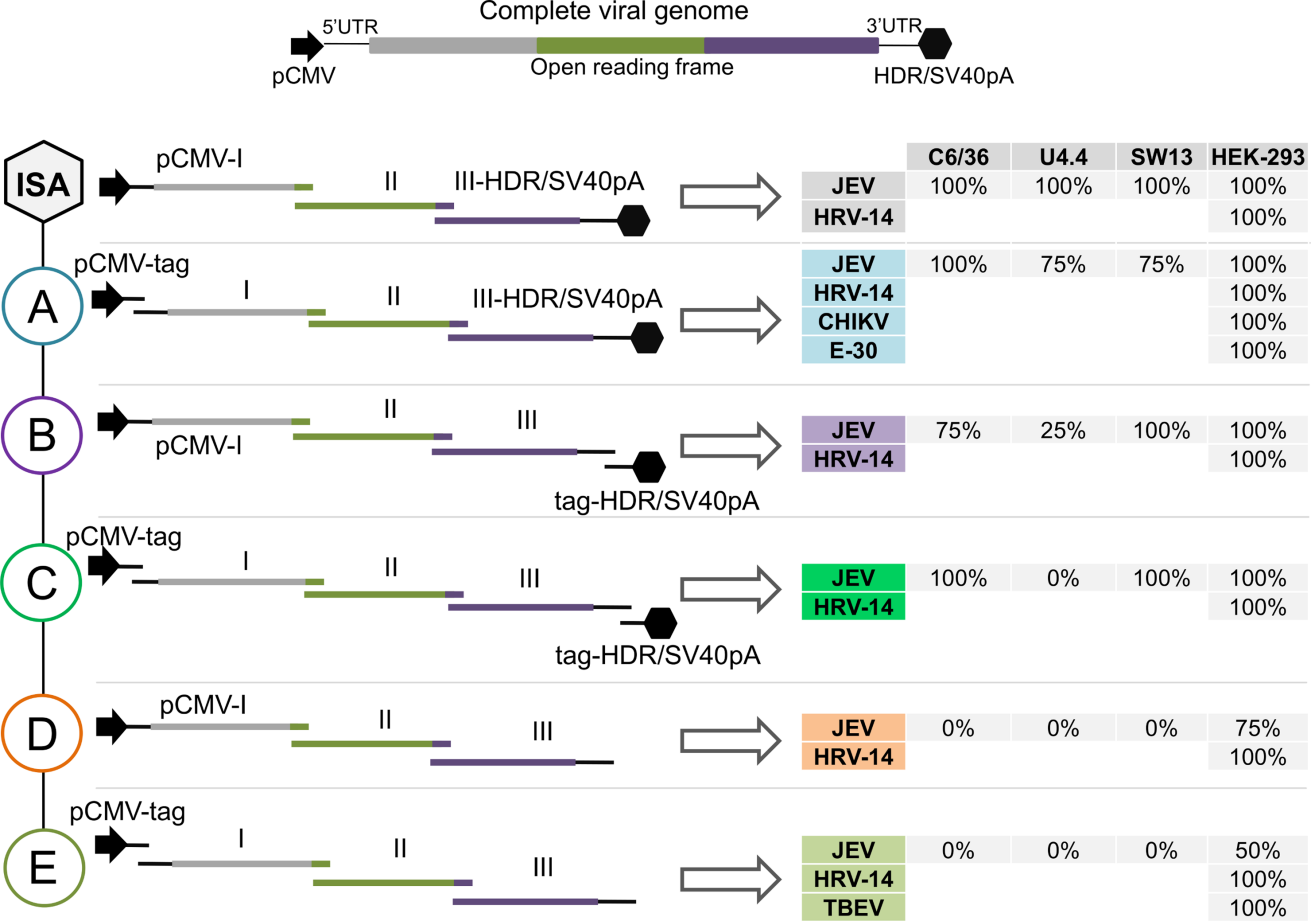
Simplified designs used to rescue infectious RNA viruses. The entire viral genome, flanked respectively at the 5’ and 3’ untranslated regions (UTRs) by the human cytomegalovirus promoter (pCMV) and the hepatitis delta ribozyme followed by the simian virus 40 polyadenylation signal (HDR/SV40pA) is schematized at the top of the figure. For each design, called A to E and the classical ISA design (named ‘ISA’), the number of overlapping subgenomic amplicons ranged from three to five (Left panel). These amplicons were amplified by PCR/RT-PCR, purified, pooled and then transfected into permissive cells. Each design was tested with a panel of viruses in mosquito (C6/36, U4.4) and mammalian (SW13, HEK-293) cell lines. After cell transfection and two serial passages, virus replication was demonstrated using a combination of several criteria (CPE, production of viral RNAs and infectious particles in cell supernatant medium). The percentage of success to recover a virus was calculated using the result of four independent experiments of each procedure and is reported (Right panel).

First, the Japanese encephalitis virus (JEV; *Flavivirus*) belonging to the *Flaviviridae* family was chosen as model to evaluate (i) the possibility to provide the pCMV and the HDR/SV40pA sequences as separate amplicons and (ii) the necessity of using the HDR/SV40pA sequence for reverse genetics of RNA viruses. To this end, five new designs (**Fig 1**) were tested using mosquito (C6/36, U4.4) and mammalian (SW13, HEK-293) cells. DNA amplicons encompassing the entire viral genome were generated by PCR or RT-PCR either from *de novo* synthesized gene or from viral RNAs as template (see **Table A** in **S1 Text** for more details).

After cell transfection with 3, 4 or 5 amplicons depending on the design tested and two serial passages, virus replication was assessed using a combination of several criteria as previously described [8–10, 12]: (i) presence of cytopathic effect (CPE), (ii) production of viral RNAs, assessed using a real-time RT-PCR assay, (iii) production of infectious particles, measured using a standard TCID_50_ assay and (iv) genome integrity verification by Next Generation Sequencing (NGS; one sample per design; only for JEV). The robustness of each procedure was evaluated using the result of four independent experiments (**Fig 1**). As expected, cytopathic effects (CPE) were always observed in positive cell cultures from the first passage, confirming the recovery of infectious viruses with amounts of viral RNAs detected ranging from 8.08 to 9.06 log_10_ copies/mL and infectious titers ranging from 5.04 to 8.01 log_10_ TCID_50_/mL (**Table 1**).

**Table 1:** Characterization of the recovered viruses. Replication of recovered viruses was assessed using a combination of several criteria: (i) the presence or absence of cytopathic effect (CPE) is highlighted in green or red, respectively, (ii) the amount of viral RNAs in cell supernatant at the second passage, assessed using a real-time RT-PCR assay is reported as mean Log_10_ copies/mL ±SD (iii) the infectious titer of each of the rescued virus at the second passage is expressed as a mean log_10_ TCID_50_/mL ±SD.

Viruses	JEV								HRV-14		CHIKV		E-30		TBEV	
Cells	C6/36		U4.4		SW13		HEK-293		HEK-293							
Haiku Designs	Viral RNAs	TCID_50_	Viral RNAs	TCID_50_	Viral RNAs	TCID_50_	Viral RNAs	TCID_50_	Viral RNAs	TCID_50_	Viral RNAs	TCID_50_	Viral RNAs	TCID_50_	Viral RNAs	TCID_50_
**ISA**	9.06 ±0.28	8.01 ±0.20	8.65 ±0.28	6.63 ±0.35	8.86 ±0.12	5.19 ±0.12	8.77 ±0.08	7.29 ±0.18	10.01 ±0.22	4.59 ±0.29	N.D.	N.D.	N.D.	N.D.	N.D.	N.D.
**A**	8.71 ±0.19	7.86 ±0.26	8.76 ±0.14	6.43 ±0.10	8.59 ±0.32	5.04 ±0.15	8.85 ±0.11	5.36 ±0.32	9.71 ±0.38	4.55 ±0.70	10.65 ±0.55	4.69 ±0.12	10.33 ±0.59	6.56 ±0.00	N.D.	N.D.
**B**	8.71 ±0.19	7.93 ±0.10	8.80 ±0.20	6.36 ±0.04	8.68 ±0.29	5.11 ±0.21	8.73 ±0.13	5.50 ±0.31	10.28 ±1.93	4.94 ±0.42	N.D.	N.D.	N.D.	N.D.	N.D.	N.D.
**C**	8.86 ±0.12	7.94 ±0.12	<3.00	<1.8^¶^	8.69 ±0.37	5.07 ±0.20	8.72 ±0.07	5.56 ±0.29	9.98 ±0.88	5.03 ±0.22	N.D.	N.D.	N.D.	N.D.	N.D.	N.D.
**D**	<3.00	<1.8^¶^	<3.00	<1.8^¶^	<3.00	<1.8^¶^	8.08 ±0.31	5.44 ±0.37	10.95 ±0.56	4.82 ±0.33	N.D.	N.D.	N.D.	N.D.	N.D.	N.D.
**E**	<3.00	<1.8^¶^	<3.00	<1.8^¶^	<3.00	<1.8^¶^	8.54 ±0.32	5.19 ±0.10	9.66 ±0.88	4.79 ±0.32	N.D.	N.D.	N.D.	N.D.	9.86 ±0.93	7.36 ±0.05

Mean ±SD of amounts of viral RNAs and infectious titers were calculated from four replicates performed at the same time.

^¶^ Detection threshold of the assay is 1.8 log_10_ TCID_50_/ml.

N.D.: Not Determined.

As previously described [8, 10], using the standard ISA design we obtained a percentage of success of 100% to recover infectious JEV particles on mammalian and mosquito cells (**Fig 1**, ISA design). Then, the pCMV sequence, tagged at its 3’-extremity by the first thirty nucleotides of the first part of the viral genome, was supplied as a separate DNA fragment, named pCMV-tag (**Fig 1**, design A). This condition lead to an elevated percentage of success, ranging between 75% and 100% with all cell lines tested. Using HEK-293 cells for transfection, we also confirmed for viruses belonging to the *Togaviridae* and *Picornaviridae* families, such as Chikungunya virus (CHIKV; *Alphavirus*) and Echovirus 30 (E-30; *Enterovirus*), that the pCMV sequence can be supplied separately (**Fig 1**, design A), leading to rescue of infectious particles with a 100% effectiveness.

When the HDR/SV40pA sequence, tagged at its 5’-extremity by the last thirty nucleotides of the last part of the viral genome, was supplied as a separate DNA fragment, named tag-HDR/SV40pA (**Fig 1**, design B), we observed with the JEV variable effectiveness depending on the cell type used: 100% with SW13 and HEK-293 cells, 75% with C6/36 cells and only 25% with U4.4 cells.

Finally, when both pCMV and HDR/SV40pA sequences, tagged at their 3’- and 5’-extremities by the first and last thirty nucleotides of the viral genome respectively, were provided as separate overlapping fragments (**Fig 1**, design C), we observed with the JEV a success of 100% with C6/36, SW13 and HEK-293 cells, but 0% success with U4.4 cells. This validates the Haiku design as a very simple and robust tool allowing viral rescue in classical mammalian and mosquito cells. Overall, this confirms also that the DNA recombination mechanisms implicated behind the ISA procedure are ancestral and present in evolutionary highly divergent cells [10]. These yet unknown mechanisms appeared not limiting to reach a full success of virus rescue, which is consistent with the possibility of increasing the number of overlapping amplicons up to 10 using the classical ISA procedure [8, 10].

Nevertheless, these results also suggested that HDR/SV40pA is required in U4.4 cells or that the efficacy of recombination between fragment III and tag-HDR/SV40pA in these cells was not sufficient to rescue the virus. Although C6/36 and U4.4 cells have been derived from the same mosquito species (*Aedes albopictus*), different results were obtained with both cell lines. The reasons that could explain these discrepancies were unclear and might rather rely on a difference of transfection efficacy.

To further evaluate the actual requirement for the presence of HDR/SV40pA, we removed it from the 3’-extremity of the last fragment, while providing pCMV fused to the fragment I (**Fig 1**, design D) or as a separate amplicon (**Fig 1**, design E). Using JEV, no virus replication was detected, except with HEK-293 cells where unexpectedly an effectiveness ranging from 50 to 75% was obtained (**Fig 1**). These results indicated that HDR/SV40pA is required in most of the cells tested, but not in HEK-293 cells, where HDR/SV40pA does not look critical to allow an efficient rescue for unknown reasons. Interestingly, this particular cell line could therefore allow implementing the simplest conceivable Haiku protocol, in which pCMV is provided separately together with unmodified genomic amplicons (**Fig 1**, design E). Accordingly, we decided to use HEK-293 cells and to apply this Haiku design to a biological sample (**Fig 1**, design E). To get closer to conditions where nucleic acids-derived clinical and/or animal samples are the only materials available, we thus used RNAs extracted from a brain suspension filtrate collected from a TBEV infected mouse as template to generate overlapping DNA fragments [12]. As expected, an efficacy of 100% was reached with this protocol.

Single-stranded positive-sense RNA viruses contain members for whom the 3’ extremity of their genomic viral RNA can be polyadenylated, (such as members of *Togaviridae* and *Picornaviridae families*), or not (such as flaviviruses). Thereby, we decided to test all the Haiku designs with a single-stranded positive-sense RNA virus belonging to the *Picornaviridae* family: the human rhinovirus type 14 (HRV-14; *Enterovirus*). Again, to get closer to conditions where nucleic acids-derived clinical and/or animal samples are the only materials available, we produced DNA amplicons by RT-PCR using viral RNAs as template, except when fragment I and III need to be fused with pCMV or HDR/SV40pA sequences. In the latter cases, *de novo* synthetized gene or a molecular clone containing the fused sequence was used as template (see **Table A** in **S1 Text** for more details). Following transfection of HEK-293 cells and serial passages in MRC-5 cells, virus replication was observed for all designs as well as for the classical ISA design with a 100% success of rescue (**Fig 1**). This illustrates the remarkable potential of HEK-293 cells to rescue viruses, and the versatility of the new simplified designs. Of note, the HDR/SV40pA sequence appears again not critical but might be explained here by the fact that HRV-14 genomic RNA is polyadenylated and that the fragment III used in conditions D and E already possesses a polyA tail of 25 As. In addition, a previous study showed that following transfection of RD cells with an enterovirus infectious clone under the control of pCMV and with or without hepatitis delta virus ribozyme in its 3’ extremity, equally infectious viruses were generated confirming that in some cases HDR/SV40pA did not play a crucial role to rescue an enterovirus [13].

In conclusion, we demonstrated here that it is possible to simplify the ISA procedure by providing the pCMV as a separate overlapping DNA fragment at least to rescue viruses from *Flaviviridae*, *Togaviridae* or *Picornaviridae* families. Regarding the HDR/SV40pA sequence, we showed that an ultimately simplified procedure in which no HDR/SV40pA will be used could be implemented for viruses having a polyadenylated 3’ extremity of their genomic RNA like those from *Togaviridae* or *Picornaviridae* families. Interestingly, concerning viruses with non-polyadenylated 3’ genomic extremity such as members of the *Flaviviridae*, if HEK-293 cells are permissive for the virus, this cell line together with the Haiku procedure providing only the pCMV as a separate overlapping DNA fragment remains the simplest rescue method. For all other cell lines, including mammalian as well as insect cells, HDR/SV40pA could be successfully provided as a separate DNA fragment.

These streamline "Haiku" designs provide an even more simple, rapid, versatile and cost-effective new tool to rescue viruses. Indeed, only the generation of overlapping amplicons encompassing the entire viral genome is now required without the need of *de novo* synthesis of pCMV and HDR/SV40pA fused to viral extremities or to perform fragment fusion by PCR. This methodology could represent a significant advance to decrease the timeframe between the emergence of a new viral agent and the vaccine seed virus production, allowing also to safely use the classical cell lines employed for vaccine production, including insect cell lines. Altogether, these Haiku variations conserve all the advantages of the standard ISA method such as its broad applicability to a wide range of viruses in different cell lines. These simplified designs give the possibility to easily study the role of some point mutations, such as resistance mutations to antiviral agents because the introduction of a mutation requires the modification of only one subgenomic DNA fragment without the need of cloning [8].

Because subgenomic amplicons can be generated using a variety of original materials including viral RNAs, the Haiku ISA design has the potential to greatly facilitate rescue of viral pathogens from clinical and/or animal samples that have been inactivated or deteriorated during collection or storage. Indeed, we previously demonstrated that low-copy samples may be used for this kind of procedure [12]. These Haiku designs also opens the door to a new concept of affordable long-term storage of viral strains in which subgenomic amplicons encompassing the entire viral genome could be used as non-infectious back-up material.

Finally, if the molecular mechanisms allowing the rescue of viruses from overlapping DNA fragments are currently unknown, this study confirms that these specific mechanisms are present and conserved into evolutionary distant cells, like insect and mammalian cells [10]. The description of these mechanisms is currently in progress and is expected to allow in the future further improvements of reverse genetics methods for RNA viruses. Especially, the minimum requirement for the overlapping region in term of length or %CG could also lead to substantial improvements in the rescue efficiency at the cell by cell basis.

## Materials and methods

### Cells

Human adrenal carcinoma SW13 cells (ATCC number CCL-105) were grown at 37°C with 5% CO_2_ in RPMI Medium (Life technologies) supplemented with 10% heat-inactivated fetal bovine serum (FBS; Life Technologies) and 1% penicillin/ streptomycin (PS; 5000U.mL^-1^ and 5000μg.mL^-1^; Life Technologies). Human embryonic kidney HEK-293 cells (ATCC number CCL-1573) were grown at 37°C with 5% CO2 in a minimal essential medium (MEM, Life Technologies) supplemented with 7% FBS, 1% PS and 1% Minimun Essential Medium Non-Essential Amino Acids (MEM NEAA; Life Technologies). Human fetal lung fibroblast MRC-5 (ATCC number CCL-171) were grown at 37°C with 5% CO_2_ in a Basal Medium Eagle (Life Technologies) supplemented with 10% FBS and 1% PS. *Aedes albopictus* C6/36 cells (ATCC number CRL-1660) and U4.4 (kindly provided by AB Failloux from Institut Pasteur, Paris) were grown at 28°C without CO_2_ in a Leibovit’z L-15 medium (Life Technologies) with 10% FBS, 5% TPB and 1% PS.

### Viruses, nucleic acid material and animal sample

Japanese encephalitis virus (JEV; *Flaviviridae*; *Flavivirus*) genotype I strain CNS769_Laos_2009 (Genbank accession number: KC196115), called JEV in the present study, was isolated in June 2009 from the cerebrospinal fluid of a patient in Laos [14]. Chikungunya virus (CHIKV; *Togaviridae*; *Alphavirus*) strain LR2006 OPY1, called CHIKV in the present study, was derived from an infectious clone previously described (Genbank accession number: DQ443544) [15]. Human rhinovirus type 14 strain 1059 (*Picornaviridae*; *Enterovirus*), called HRV-14 in the present study, was derived from the pWR3.26 infectious clone (ATCC VRMC-7; Genbank accession number: L05355)[16]. A modified version of pWR3.26, named HRV-14 I.C., containing the pCMV and the HDR/SV40pA sequence at the 5’ and 3’ extremities of the full length viral genome respectively, was generated as described in **Supplementary Methods A** in **S1 Text**. Echovirus 30 strain 4-MRS2013 (*Picornaviridae*; *Enterovirus*), called E-30 in the present study, was isolated in Marseille in 2013 during an outbreak of aseptic meningitis (Genbank accession number: KF920600)[17].

We used a mouse brain suspension filtrate collected from a mouse infected by the tick-borne encephalitis virus (TBEV; *Flaviviridae*; *Flavivirus*) strain Oshima 5–10 as previously described [12]. Briefly, this sample was collected from a five-week-old C57/Bl6 female mouse inoculated intraperitoneally with 2.10^6^ TCID50 of TBEV and euthanized 7 days later. Animal experiments were approved by the Ethics Committee “Marseille-C2EA-14” (protocol number #2504) and performed in compliance with French and European regulations (directive 210/63/EU).

### Preparation of DNA fragments

The origin of the initial material used to generate the following subgenomic amplicons is summarized in **Table A** in **S1 Text**.

The fragment named pCMV-tag corresponded to the pCMV sequence tagged at its 3’ extremity by the first thirty nucleotides of the viral genome (**Fig 1**). It was amplified by PCR from a *de novo* synthesized DNA plasmid previously described [8], named DNS-I Dengue, which contain the pCMV followed by the first third portion of the dengue virus type 4 (DENV-4) strain Dak HD 34 460 genome. The reverse primer used for amplification was tagged by the 30 first nucleotides of the viral genome of the corresponding virus.

The fragment named pCMV-I corresponded to the pCMV fused to the first part of the viral genome **(Fig 1**). It was amplified by PCR using as template a *de novo* synthesized DNA plasmid [8],which contain the pCMV followed by the first third portion of JEV or the infectious clone named HRV-14 I.C. (HRV-14).

The fragments named I, II and III corresponded to the first, second and third part of the complete viral genome (**Fig 1**). For TBEV all fragments were amplified by RT-PCR using as template extracted nucleic acids from brain suspension filtrate. For JEV, fragment II was amplified by PCR using as template a *de novo* synthesized DNA plasmid previously described [8], which contain corresponding region of JEV genome, and fragment I and III were amplified by RT-PCR using as template the extracted nucleic acids from infectious cell supernatant media. For HRV14, CHIKV and E-30, the fragments named I, II and III were amplified by RT-PCR using as template the extracted nucleic acids from infectious cell supernatant media.

The fragment named III-HDR/SV40pA corresponded to the last part of the viral genome followed by the HDR/SV40pA (**Fig 1**). It was amplified by PCR using as template a *de novo* synthesized DNA plasmid [8], which contain last part of the JEV genome followed by HDR/SV40pA at its 3’ extremity or an infectious clone for HRV-14 (HRV-14 I.C.) and CHIKV. For E-30, it was amplified by PCR in two steps as previously described [12]: the 39 final nucleotides of the viral genome, the polyA tail and the HDR/SV40pA were synthesized *de novo* (Genscript) and then merged by fusion PCR with the pre-amplified fragment III.

The fragment named tag-HDR/SV40pA corresponded to the HDR/SV40pA and was tagged at its 5’ extremity by the last thirty nucleotides of the viral genome (**Fig 1**). It was amplified by PCR from the CHIKV infectious clone. The forward primer used for amplification was tagged by the 30 final nucleotides of the viral genome of the corresponding virus.

### Amplification of DNA fragments

When a DNA template was used (*de novo* synthesized DNA or infectious clone), amplicons were generated by PCR using the Platinum PCR SuperMix High Fidelity kit (Life Technologies) and a Biometra TProfessional Standard Gradient thermocycler as previously described [8]. When a RNA template was used (nucleic acid extract from cell supernatant medium or mice brain suspension filtrate), amplicons were generated by RT-PCR using the Superscript III One-Step RT-PCR Platinum Taq High Fidelity kit (Life Technologies) and a Biometra TProfessional Standard Gradient thermocycler as previously described [8]. Primer sequences are detailed in **Table B** in **S1 Text**.

Size of the PCR products was verified by gel electrophoresis and purifications performed using the Amicon Ultra 0.5 ml kit (Millipore). When infectious clones were used as template, a digestion step with the restriction enzyme DpnI (New England Biolabs) was performed to remove the template. To control the efficiency of this additional step we transfected as a negative control an incomplete equimolar mix of the DNA fragments (without fragment II, 3μg final). This control did not produce any infectious virus as previously described [8].

### Cell transfection

An equimolar mixture of the amplified DNA fragments was used to transfect 12.5cm^2^ culture flask of mammalian or mosquito cells as previously described [9]. Briefly, DNA-lipid complex containing 3μg of DNA and 6μL of P3000 reagent diluted in a total volume of 500 μL of Opti-MEM medium was added to a 12.5 cm^2^ culture flask of subconfluent cells which contains 1 mL of medium without antibiotics. After an incubation period of 12 hours at 37°C with 5% CO_2_, cells were washed twice (HBSS; Life Technologies) and 3 mL of medium with antibiotic were added. Cells were then incubated 7 days at standard conditions (28°C for mosquito cells, 33°C with 5% CO_2_ for HRV-14 expression and 37°C with 5% CO_2_ for all other cases). Each experiment was performed in quadruplicate at the same time. Cell supernatant media were then serially passaged twice using the same cell types for all viruses except for the HRV14, for which MRC-5 cells were used and incubated at 33°C with 5% CO_2_. For each passage, 333μl of cell supernatant clarified by centrifugation were inoculated into a new 12.5 cm^2^ culture flask of confluent cells. After an incubation period of 2 hours, cells were washed twice (HBSS) and 3mL of fresh medium was added before incubation (2–6 days depending of the viruses) at standard conditions.

### Nucleic acid extraction

Extraction of nucleic acid was performed using the EZ1 advanced XL machine (Qiagen) with the EZ1 Virus Mini Kit v2.0 (Qiagen).

### Real-time quantitative RT-PCR assay

Relative quantification of viral RNA was performed using the GoTaq probe-1-step RT-qPCR system kit (Promega). Primer and probe sequences are detailed in **Table C** in **S1 Text**. The mixture (final volume: 20 μl) contained 10μL of Gotaq probe qPCR Master Mix, 0.5 μL of each primer (10μM working solution were used), 0.2μL of probe (10μM working solution were used), 0.5 μL of Go script RT mix, 0.3 μL of nuclease-free water and 8μL of extracted nucleic acids. Assays were performed using the CFX96 Touch real-time PCR machine (Bio-Rad) with the following conditions: 50°C for 15 min, 95°C for 2 min, followed by 45 cycles of 95°C for 15 s, 60°C for 40 s. Data collection occurred during the 60°C step. The quantity of viral RNA expressed as copies/mL was calculated from standard curves using synthetic RNA.

### Tissue culture infectious dose 50 (TCID50) assay

For each determination, a 96-well plate culture of confluence cells was used. C6/36 cells were used for JEV, MRC-5 cells were used for HRV14 and HEK were used for CHIKV, TBEV and E-30. A 100μL of medium per well was inoculated with 50μL of serial 10-fold dilutions of clarified supernatant medium. Each row included 6 wells of the same dilution and two negative controls. The plates were incubated for 7 days and read for absence or presence of CPE in each well. The determination of the TCID_50_/mL was performed using the method of Reed and Muench [18].

### Sequence analysis of complete genomes

Sequencing of complete genomes was performed as previously described [9, 12] using the Ion PGM Sequencer (Life Technologies) and analyses were conducted using the CLC Genomics Workbench 6 software. Genome integrity of the sequenced genomes was considered as verified when the nucleotides similarity was ≥99.9%.

## Supporting information

S1 Text Supplemental data. **Table A:** Origin of the initial material used to generate subgenomic amplicons by PCR/RT-PCR
**Table B:** Primers used to obtain DNA fragments described by PCR/RT-PCR
**Table C:** Primers and probes used for the quantitative real-time RT-PCR assays
**Table D:** Primers used for HRV-14 infectious clone construction
**Supplementary Methods A:** pWR3.26 plasmid modifications
(PDF)

## References

[1] Pijlman GP. Enveloped virus-like particles as vaccines against pathogenic arboviruses. Biotechnology journal. 2015;10(5):659–70. doi: 10.1002/biot.201400427 . 25692281 10.1002/biot.201400427

[2] Aliota MT, Bassit L, Bradrick SS, Cox B, Garcia-Blanco MA, Gavegnano C, et al Zika in the Americas, year 2: What have we learned? What gaps remain? A report from the Global Virus Network. Antiviral research. 2017 doi: 10.1016/j.antiviral.2017.06.001 . 28595824 10.1016/j.antiviral.2017.06.001 PMC5920658

[3] Mire CE, Matassov D, Geisbert JB, Latham TE, Agans KN, Xu R, et al Single-dose attenuated Vesiculovax vaccines protect primates against Ebola Makona virus. Nature. 2015;520(7549):688–91. doi: 10.1038/nature14428 ; PubMed Central PMCID: PMCPMC4629916. 25853476 10.1038/nature14428 PMC4629916

[4] Bradley T, Pollara J, Santra S, Vandergrift N, Pittala S, Bailey-Kellogg C, et al Pentavalent HIV-1 vaccine protects against simian-human immunodeficiency virus challenge. Nature communications. 2017;8 ARTN 15711 doi: 10.1038/ncomms15711 WOS:000402869200002. 28593989 10.1038/ncomms15711 PMC5472724

[5] Sissoko D, Laouenan C, Folkesson E, M'Lebing AB, Beavogui AH, Baize S, et al Experimental Treatment with Favipiravir for Ebola Virus Disease (the JIKI Trial): A Historically Controlled, Single-Arm Proof-of-Concept Trial in Guinea. PLoS medicine. 2016;13(3). ARTN e1001967 doi: 10.1371/journal.pmed.1001967 WOS:000373039400002. 26930627 10.1371/journal.pmed.1001967 PMC4773183

[6] Conzelmann KK. Reverse genetics of mononegavirales. Curr Top Microbiol Immunol. 2004;283:1–41. . 15298166 10.1007/978-3-662-06099-5_1

[7] Aubry F, Nougairede A, Gould EA, de Lamballerie X. Flavivirus reverse genetic systems, construction techniques and applications: a historical perspective. Antiviral research. 2015;114:67–85. doi: 10.1016/j.antiviral.2014.12.007 . 25512228 10.1016/j.antiviral.2014.12.007 PMC7173292

[8] Aubry F, Nougairede A, de Fabritus L, Querat G, Gould EA, de Lamballerie X. Single-stranded positive-sense RNA viruses generated in days using infectious subgenomic amplicons. The Journal of general virology. 2014;95(Pt 11):2462–7. Epub 2014/07/24. doi: 10.1099/vir.0.068023-0 ; PubMed Central PMCID: PMCPmc4202267. 25053561 10.1099/vir.0.068023-0 PMC4202267

[9] Atieh T, Baronti C, de Lamballerie X, Nougairede A. Simple reverse genetics systems for Asian and African Zika viruses. Scientific reports. 2016;6:39384 doi: 10.1038/srep39384 ; PubMed Central PMCID: PMC5171905. 27991555 10.1038/srep39384 PMC5171905

[10] Atieh T, Nougairede A, Klitting R, Aubry F, Failloux AB, de Lamballerie X, et al New reverse genetics and transfection methods to rescue arboviruses in mosquito cells. Scientific reports. 2017;7(1):13983 doi: 10.1038/s41598-017-14522-6 ; PubMed Central PMCID: PMCPMC5656662. 29070887 10.1038/s41598-017-14522-6 PMC5656662

[11] de Fabritus L, Nougairede A, Aubry F, Gould EA, de Lamballerie X. Utilisation of ISA Reverse Genetics and Large-Scale Random Codon Re-Encoding to Produce Attenuated Strains of Tick-Borne Encephalitis Virus within Days. PloS one. 2016;11(8):e0159564 Epub 2016/08/23. doi: 10.1371/journal.pone.0159564 ; PubMed Central PMCID: PMCPmc4993482. 27548676 10.1371/journal.pone.0159564 PMC4993482

[12] Aubry F, Nougairede A, de Fabritus L, Piorkowski G, Gould EA, de Lamballerie X. "ISA-Lation" of Single-Stranded Positive-Sense RNA Viruses from Non-Infectious Clinical/Animal Samples. PloS one. 2015;10(9):e0138703 Epub 2015/09/26. doi: 10.1371/journal.pone.0138703 ; PubMed Central PMCID: PMCPmc4583506. 26407018 10.1371/journal.pone.0138703 PMC4583506

[13] Tan CW, Tee HK, Lee MH, Sam IC, Chan YF. Enterovirus A71 DNA-Launched Infectious Clone as a Robust Reverse Genetic Tool. PloS one. 2016;11(9):e0162771 doi: 10.1371/journal.pone.0162771 ; PubMed Central PMCID: PMCPMC5019408. 27617744 10.1371/journal.pone.0162771 PMC5019408

[14] Aubry F, Vongsouvath M, Nougairede A, Phetsouvanh R, Sibounheuang B, Charrel R, et al Complete Genome of a Genotype I Japanese Encephalitis Virus Isolated from a Patient with Encephalitis in Vientiane, Lao PDR. Genome announcements. 2013;1(1). Epub 2013/03/08. doi: 10.1128/genomeA.00157-12 ; PubMed Central PMCID: PMCPmc3587933. 23469339 10.1128/genomeA.00157-12 PMC3587933

[15] Nougairede A, De Fabritus L, Aubry F, Gould EA, Holmes EC, de Lamballerie X. Random codon re-encoding induces stable reduction of replicative fitness of Chikungunya virus in primate and mosquito cells. PLoS Pathog. 2013;9(2):e1003172 doi: 10.1371/journal.ppat.1003172 ; PubMed Central PMCID: PMC3578757. 23436995 10.1371/journal.ppat.1003172 PMC3578757

[16] Lee WM, Monroe SS, Rueckert RR. Role of maturation cleavage in infectivity of picornaviruses: activation of an infectosome. Journal of virology. 1993;67(4):2110–22. ; PubMed Central PMCID: PMCPMC240305. 8383233 10.1128/jvi.67.4.2110-2122.1993 PMC240305

[17] Nougairede A, Bessaud M, Thiberville SD, Piorkowski G, Ninove L, Zandotti C, et al Widespread circulation of a new echovirus 30 variant causing aseptic meningitis and non-specific viral illness, South-East France, 2013. Journal of clinical virology: the official publication of the Pan American Society for Clinical Virology. 2014;61(1):118–24. Epub 2014/06/29. doi: 10.1016/j.jcv.2014.05.022 . 24973284 10.1016/j.jcv.2014.05.022

[18] REED LJ, MUENCH H. A SIMPLE METHOD OF ESTIMATING FIFTY PER CENT ENDPOINTS. American Journal of Epidemiology. 1938;27(3):493–7.

