# Supplementary material for: Haiku: New paradigm for the reverse genetics of emerging RNA viruses
Source: PLoS One. 2018 Feb 13;13(2):e0193069. doi: 10.1371/journal.pone.0193069 (PMC5811033; doi:10.1371/journal.pone.0193069)
Supplement: S1 Text — Table A: Origin of the initial material used to generate subgenomic amplicons by PCR/RT-PCR Table B: Primers used to obtain DNA fragments described by PCR/RT-PCR Table C: Primers and probes used for the quantitative real-time RT-PCR assays Table D: Primers used for HRV-14 infectious clone construction Supplementary Methods A: pWR3.26 plasmid modifications (PDF) [file pone.0193069.s001.pdf]

S1 Text. Supplemental data

Haiku: new paradigm for the reverse genetics of RNA viruses

Thérèse Atieh, Miriam Diala El Ayoubi, Fabien Aubry, Stéphane Priet, Xavier de Lamballerie

and Antoine Nougairède

Table A: Origin of the initial material used to generate subgenomic amplicons by PCR/RT-PCR
DNS and I.C. mean *de novo* synthesis and infectious clone respectively. pCMV-I, pCMV-tag, I, II, III, III-HDR/SV40pA and Tag-HDR/SV40pA refer to the name of the amplicons described in fig 1.

<table>
<tr><th>Designs</th><th>Nb of amplicons</th><th>Viruses</th><th colspan="5">Origin of initial material</th></tr>
<tr><td rowspan="3">ISA</td><td rowspan="3">3</td><td></td><td>pCMV-I</td><td>II</td><td>III-HDR/SV40pA</td><td></td><td></td></tr>
<tr><td>JEV</td><td>DNS-I JEV</td><td>DNS-II JEV</td><td>DNS-III JEV</td><td></td><td></td></tr>
<tr><td>HRV-14</td><td>HRV-14 I.C.</td><td>Viral RNA</td><td>HRV-14 I.C.</td><td></td><td></td></tr>
<tr><td rowspan="5">A</td><td rowspan="5">4</td><td></td><td>pCMV-tag</td><td>I</td><td>II</td><td>III-HDR/SV40pA</td><td></td></tr>
<tr><td>JEV</td><td rowspan="4">DNS-I Dengue</td><td rowspan="4">Viral RNA</td><td>DNS-II JEV</td><td>DNS-III JEV</td><td></td></tr>
<tr><td>HRV-14</td><td rowspan="3">Viral RNA</td><td>HRV-14 I.C.</td><td></td></tr>
<tr><td>CHIKV</td><td>CHIKV I.C.</td><td></td></tr>
<tr><td>E-30</td><td>Viral RNA + DNS E-30</td><td></td></tr>
<tr><td rowspan="3">B</td><td rowspan="3">4</td><td></td><td>pCMV-I</td><td>II</td><td>III</td><td>Tag-HDR/SV40pA</td><td></td></tr>
<tr><td>JEV</td><td>DNS-I JEV</td><td>DNS-II JEV</td><td>Viral RNA</td><td>CHIKV I.C.</td><td></td></tr>
<tr><td>HRV-14</td><td>HRV-14 I.C.</td><td colspan="2">Viral RNA</td><td>CHIKV I.C.</td><td></td></tr>
<tr><td rowspan="3">C</td><td rowspan="3">5</td><td></td><td>pCMV-tag</td><td>I</td><td>II</td><td>III</td><td>Tag-HDR/SV40pA</td></tr>
<tr><td>JEV</td><td rowspan="2">DNS-I Dengue</td><td rowspan="2">Viral RNA</td><td>DNS-II JEV</td><td rowspan="2">Viral RNA</td><td rowspan="2">CHIKV I.C.</td></tr>
<tr><td>HRV-14</td><td>Viral RNA</td></tr>
<tr><td rowspan="3">D</td><td rowspan="3">3</td><td></td><td>pCMV-I</td><td>II</td><td>III</td><td></td><td></td></tr>
<tr><td>JEV</td><td>DNS-I JEV</td><td>DNS-II JEV</td><td>Viral RNA</td><td></td><td></td></tr>
<tr><td>HRV-14</td><td>HRV-14 I.C.</td><td colspan="2">Viral RNA</td><td></td><td></td></tr>
<tr><td rowspan="4">E</td><td rowspan="4">4</td><td></td><td>pCMV-tag</td><td>I</td><td>II</td><td>III</td><td></td></tr>
<tr><td>JEV</td><td rowspan="3">DNS-I Dengue</td><td rowspan="2">Viral RNA</td><td>DNS-II JEV</td><td>Viral RNA</td><td></td></tr>
<tr><td>HRV-14</td><td colspan="2">Viral RNA</td><td></td></tr>
<tr><td>TBEV</td><td colspan="3">Viral RNA</td><td></td></tr>
</table>

Table B: Primers used to obtain DNA fragments described by PCR/RT-PCR

Primers located respectively at the 5’ and 3’ terminus of the pCMV (¶) and the HDR/SV40pA (£). Primers located at the 3’extremity of pCMV and extended by the 30 first nucleotides of the viral genome (◊). Primers located at the 5’extremity of HDR/SV40pA and extended by the 30 last nucleotides of the viral genome (□).

*All positions based on complete viral strain sequence

**Designs**	**DNA fragment**					
**JEV**	**Name**	**Length (bp)**	**Primer Forward**	**Position***	**Primer Reverse**	**Position ***
ISA	pCMV-I	4815	CACCCAACTGATCTTCAGCATCT	¶	GAAGAATGATTCTGTAAGTGTCCAG	4032-4057
	II	3003	CGTTGCCATGCCAATCTTAGCG	3980-4002	GGTGCTTGCGTCCTTCCACCAA	6961-6983
	III-HDR/SV40pA	4247	CAAATGAGTATGGAATGCTGGAAAA	6910-6935	CTCAGGGTCAATGCCAGCGCTT	£
A	pCMV-tag	789	CACCCAACTGATCTTCAGCATCT	¶	TAAGCCAAGAAGTTCACACAGATAAACTTCTCGGTTCA CTAAACGAGCTCTGC	◊
	I	4057	AGAAGTTTATCTGTGTGAACTTCT	0-24	GAAGAATGATTCTGTAAGTGTCCAG	4032-4057
	II	3003	CGTTGCCATGCCAATCTTAGCG	3980-4002	GGTGCTTGCGTCCTTCCACCAA	6961-6983
	III-HDR/SV40pA	4247	CAAATGAGTATGGAATGCTGGAAAA	6910-6935	CTCAGGGTCAATGCCAGCGCTT	£
B	pCMV-I	4815	CACCCAACTGATCTTCAGCATCT	¶	GAAGAATGATTCTGTAAGTGTCCAG	4032-4057
	II	3003	CGTTGCCATGCCAATCTTAGCG	3980-4002	GGTGCTTGCGTCCTTCCACCAA	6961-6983
	III	4055	CAAATGAGTATGGAATGCTGGAAAA	6910-6935	AGATCCTGTGTTCTTCCTCACC	10943-10965
	tag-HDR/SV40pA	224	GTAGCTGGTGGTGAGGAAGAACACAGGATCTGGCCGGCATGGTCCC AGC	□	CTCAGGGTCAATGCCAGCGCTT	£
C	pCMV-tag	789	CACCCAACTGATCTTCAGCATCT	¶	TAAGCCAAGAAGTTCACACAGATAAACTTCTCGGTTCA CTAAACGAGCTCTGC	◊
	I	4057	AGAAGTTTATCTGTGTGAACTTCT	0-24	GAAGAATGATTCTGTAAGTGTCCAG	4032-4057
	II	3003	CGTTGCCATGCCAATCTTAGCG	3980-4002	GGTGCTTGCGTCCTTCCACCAA	6961-6983
	III	4055	CAAATGAGTATGGAATGCTGGAAAA	6910-6935	AGATCCTGTGTTCTTCCTCACC	10943-10965
	tag-HDR/SV40pA	224	GTAGCTGGTGGTGAGGAAGAACACAGGATCTGGCCGGCATGGTCCC AGC	□	CTCAGGGTCAATGCCAGCGCTT	£
D	pCMV-I	4815	CACCCAACTGATCTTCAGCATCT	¶	GAAGAATGATTCTGTAAGTGTCCAG	4032-4057

	II	3003	CGTTGCCATGCCAATCTTAGCG	3980-4002	GGTGCTTGCGTCCTTCCACCAA	6961-6983
	III	4055	CAAATGAGTATGGAATGCTGGAAAA	6910-6935	AGATCCTGTGTTCTTCCTCACC	10943-10965
E	pCMV-tag	789	CACCCAACTGATCTTCAGCATCT	¶	TAAGCCAAGAAGTTCACACAGATAAACTTCTCGGTTCA CTAAACGAGCTCTGC	◊
	I	4057	AGAAGTTTATCTGTGTGAACTTCT	0-24	GAAGAATGATTCTGTAAGTGTCCAG	4032-4057
	II	3003	CGTTGCCATGCCAATCTTAGCG	3980-4002	GGTGCTTGCGTCCTTCCACCAA	6961-6983
	III	4055	CAAATGAGTATGGAATGCTGGAAAA	6910-6935	AGATCCTGTGTTCTTCCTCACC	10943-10965
**HRV-14**						
ISA	pCMV-I	3089	TCGACTCTAGAGGATCTCGAT	¶	GAGTGCAACAGTCTGTGAGATA	2301-2323
	II	2534	CGACCGGCCAGGTCTACTTA	2222-2242	AGATGGTTGATGGCAATCTT	4736-4756
	III-HDR/SV40pA	2806	CTTGAATCCTGAAGCTTTAGTCA	4623-4646	CTCAGGGTCAATGCCAGCGCTT	£
A	pCMV-tag	812	GAATAAGGGCGACACGGAAATGT	¶	CGAATGGTGGGATACCCATCCGCTGTTTTAACGGTTCA CTAAACGAGCTCT	◊
	I	2323	TTAAAACAGCGGATGGGTAT	0-20	GAGTGCAACAGTCTGTGAGATA	2301-2323
	II	2534	CGACCGGCCAGGTCTACTTA	2222-2242	AGATGGTTGATGGCAATCTT	4736-4756
	III-HDR/SV40pA	2806	CTTGAATCCTGAAGCTTTAGTCA	4623-4646	CTCAGGGTCAATGCCAGCGCTT	£
B	pCMV-I	3089	TCGACTCTAGAGGATCTCGAT	¶	GAGTGCAACAGTCTGTGAGATA	2301-2323
	II	2534	CGACCGGCCAGGTCTACTTA	2222-2242	AGATGGTTGATGGCAATCTT	4736-4756
	III	2614	CTTGAATCCTGAAGCTTTAGTCA	4623-4646	TTTTTTTTTTTTTTTTTTTTTTTTTATAAACTCCTACTTCTA CTC	7193-7237
	tag-HDR/SV40pA	217	AAAAAAAAAAAAAAAAAAAAAAAAAGGCCGGCATGGTCCCAGCCT	□	CTCAGGGTCAATGCCAGCGCTT	£
C	pCMV-tag	812	GAATAAGGGCGACACGGAAATGT	¶	CGAATGGTGGGATACCCATCCGCTGTTTTAACGGTTCA CTAAACGAGCTCT	◊
	I	2323	TTAAAACAGCGGATGGGTAT	0-20	GAGTGCAACAGTCTGTGAGATA	2301-2323
	II	2534	CGACCGGCCAGGTCTACTTA	2222-2242	AGATGGTTGATGGCAATCTT	4736-4756
	III	2614	CTTGAATCCTGAAGCTTTAGTCA	4623-4646	TTTTTTTTTTTTTTTTTTTTTTTTTATAAACTCCTACTTCTA CTC	7193-7237
	tag-HDR/SV40pA	217	AAAAAAAAAAAAAAAAAAAAAAAAAGGCCGGCATGGTCCCAGCCT	□	CTCAGGGTCAATGCCAGCGCTT	£
D	pCMV-I	3089	TCGACTCTAGAGGATCTCGAT	¶	GAGTGCAACAGTCTGTGAGATA	2301-2323

	II	2534	CGACCGGCCAGGTCTACTTA	2222-2242	AGATGGTTGATGGCAATCTT	4736-4756
	III	2614	CTTGAATCCTGAAGCTTTAGTCA	4623-4646	TTTTTTTTTTTTTTTTTTTTTTTTTATAAACTCCTACTTCTA CTC	7193-7237
E	pCMV-tag	812	GAATAAGGGCGACACGGAAATGT	¶	CGAATGGTGGGATACCCATCCGCTGTTTTAACGGTTCA CTAAACGAGCTCT	◊
	I	2323	TTAAAACAGCGGATGGGTAT	0-20	GAGTGCAACAGTCTGTGAGATA	2301-2323
	II	2534	CGACCGGCCAGGTCTACTTA	2222-2242	AGATGGTTGATGGCAATCTT	4736-4756
	III	2614	CTTGAATCCTGAAGCTTTAGTCA	4623-4646	TTTTTTTTTTTTTTTTTTTTTTTTTATAAACTCCTACTTCTA CTC	7193-7237
**CHIKV**						
A	pCMV-tag	789	CACCCAACTGATCTTCAGCATCT	¶	TAAGCCAAGAAGTTCACACAGATAAACTTCTCGGTTCA CTAAACGAGCTCTGC	◊
	I	4057	AGAAGTTTATCTGTGTGAACTTCT	0-24	GAAGAATGATTCTGTAAGTGTCCAG	4032-4057
	II	3003	CGTTGCCATGCCAATCTTAGCG	3980-4002	GGTGCTTGCGTCCTTCCACCAA	6961-6983
	III-HDR/SV40pA	4247	CAAATGAGTATGGAATGCTGGAAAA	6910-6935	CTCAGGGTCAATGCCAGCGCTT	£
**E-30**						
A	pCMV-tag	789	GAATAAGGGCGACACGGAAATGT	¶	TGGGTGGGAGCAACCCACAGGCTGTTTTAACGGTTCA CTAAACGAGCTCT	◊
	I	2277	TTAAAACAGCCTGTGGGTTGCTC	0-24	TCGCTGGTAACATATCTGTAGT	2256-2277
	II	3239	GGAAAGATGCCATGCTCGGGA	2166-2186	TTCAAATGCTGGACCTTGCACT	5383-5404
	III-HDR/SV40pA	2394	ATCAGTGGCTGGCATAATATACA	5272-5294	CTCAGGGTCAATGCCAGCGCTT	£
**TBEV**						
E	pCMV-tag	789	GAATAAGGGCGACACGGAAATGT	¶	AAGCAAACGCATGCACGTGCAAGAAAATCTCGGTTCA CTAAACGAGCTCTGC	◊
	I	4054	AGATTTTCTTGCACGTGCAT	0-20	GCCACGCCCAGGAAGAGCATGA	4033-4054
	II	4160	CTGGGATTGCCAAGCGAGG	3866-3885	CAACCCAGGCTTGTCACCATCTTT	8003-8026
	III	3166	GCAGCTTCTCGACCGGCTGTCATC	7935-7959	AGCGGGTGTTTTTCCGAGTC	11080-11100

Table C: Primers and probes used for the quantitative real-time RT-PCR assays

Virus	Position	Length	Primer Forward	Probe	Primer Reverse
**TBEV**	10236-10338	102	GCAGAGTGGGCCAGGAACAT	TCGGACAAGAGAAGTTCAAGGACT	TCCTGCATGGATCGGCATGAC
**CHIKV**	2631-2810	179	TGACCGCCATTGTGTCATCGTTG	CTGGAGACCTCGTGTTAACGTGCTTCAG	GACCTCGTATCCACGATAGTCA
**E-30**	456-601	145	CCCCTGAATGCGGCTAATCC	GGACACCCAAAGTAGTCGGTTCC	ATTGTCACCATAAGCAGCCA
**JEV**	158-217	59	ACCCCGCGTATTCCCACTA	TGGGAGTGAAGAGGG	GCCGTCCAACAAGCTCATTAC
**HRV-14**	368-572	204	AGCCTGCGTGGCGGCC	CTCCGGCCCCTGAATGCGGCTAA	GAAACACGGACACCCAAAGTAGT

Table D: Primers used for HRV-14 infectious clone construction

Primer Name	Primer Sequence
P1 F	CGGATGCCGGGAGCAGACAA
CMV tag R	ATGGCTAATGGCCAATATTGAATTTCGCGGGATCGAGATCCT
CMV tag F	AGGATCTCGATCCCGCGAAAT TCAATATTGGCCATTAGCCAT
CMV ext R	GCGCATGTGTCAATGCTTCCT
P2 F	GATTGTACTGAGAGTGCACCAT
CMV fus R	CGGTTCACTAAACGAGCTCT
CMV ext F	GAATAAGGGCGACACGGAAATGT
CMV tag HRV14 R	GATACCCATCCGCTGTTTTAACGGTTCACTAAACGAGCTCT
CMV tag HRV14 F	AGAGCTCGTTTAGTGAACCGTTAAAACAGCGGATGGGTATC
HRV 1R	AGCATGAGATCTTTAACTGGTT
CMV fus F	TCAATATTGGCCATTAGCCAT
HRV 2R	TTA GAT GGG TCC ATT GAC AGT GAT
P3 F	CCAATAGGCCGAAATCGGCAA
HRV 3R	GGCAGTGGGTACAGCCTATA

Supplemental Methods: pWR3.26 plasmid modifications

All the following constructions were verified by full-genome sequencing. Primers are detailed in **Table D** in **S1 Text**.

First, a molecular clone of HRV14 under the control of the CMV promoter (pCMV) and harboring a 42(A)-tail was generated by PCR using the Platinum PCR SuperMix High Fidelity kit (Life Technologies) by the replacement of the T7 promoter in pWR3.26 by pCMV. Briefly, a pWR3.26 plasmid backbone region upstream of the T7 promoter and containing the Sal I restriction site was amplified by PCR using a forward primer P1F and the reverse primer CMV tag R extended with the first twenty nucleotides of the pCMV. The pCMV was amplified from a *de novo* synthesized DNA plasmid previously described[8], named DNS-I Dengue, which contain the pCMV followed by the 5' extremity of the genome of the dengue virus type 4 (DENV-4) strain Dak HD 34 460 amplified using a forward primer CMV tag F extended with the last twenty nucleotides of the above amplicon and a reverse primer CMV ext R. These two amplicons were then merged by fusion PCR to obtain the F1 fragment using P2 F and CMV fus R. Similarly, another pCMV amplicon was obtained using the primers CMV tag F and CMV tag HRV14 R. An amplicon containing the beginning of the HRV14 genome and containing the Nar I restriction site was amplified using the CMV tag HRV14 F and HRV 1R. Then, a second fragment F2 was obtained after the fusion of the two latter amplicons using the primers CMV fus F and HRV 2R. The F1 and F2 fragments were fused using the primers P3 F and HRV 3R to generate a final F3 fragment, which was then digested by the restriction enzymes SalI and NarI. This fragment was cloned back into the digested pWR3.26 to generate the resulting infectious clone pWR3.26-pCMV.

Then, the molecular clone of HRV14 under the control of pCMV and harboring a 25(A)-tail followed by a hepatitis delta ribozyme and the simian virus 40 polyadenylation signal, named HRV14 I.C., was generated as follow. A synthetic DNA fragment, surrounded by the restriction sites SphI and MluI, that includes the last 630 bp of the HRV14 genome and followed by the hepatitis delta ribozyme and the simian virus 40 polyadenylation signal sequence was ordered from Genscript. The fragment was cloned

back into the SphI and MluI (New England Biolabs) digested pWR3.26-pCMV to generate the resulting infectious clone HRV14 I.C.
